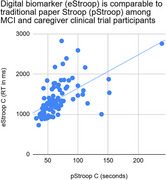# Digital biomarker for assessing executive function in a remote iPACES clinical trial for mild cognitive impairment: The comparability of the electronic Stroop vs. the paper Stroop

**DOI:** 10.1002/alz70857_107788

**Published:** 2025-12-26

**Authors:** Cay Anderson‐Hanley, Nelson A. Roque, Josh Rogers, Valerie Needham, Stella Panos, Gary Warner

**Affiliations:** ^1^ Neuroscience Program, Union College, Schenectady, NY, USA; ^2^ iPACES, Clifton Park, NY, USA; ^3^ The Pennsylvania State University, University Park, PA, USA; ^4^ UCLA, Los Angeles, CA, USA; ^5^ University of Rochester, Rochester, NY, USA

## Abstract

**Background:**

Clinical trials to ameliorate mild cognitive impairment (MCI) and stave off Alzheimer's and related dementias (ADRDs) can benefit from digital biomarkers that can efficiently evaluate baseline cognitive functioning and can detect change over time to evaluate intervention effects. Prior research has examined the comparability of standard paper neuropsychological assessments with a variety of digital tools, initially “computerized” or “electronic” tasks, now also described as “screen‐based,” “mobile” or “smart phone” tasks. When compiling the neuropsychological protocol for the pedal‐n‐play neuro‐exergaming clinical trial for MCI (iPACES: interactive Physical and Cognitive Exercise System), a fully remote national trial launching at the outset of the COVID pandemic, we considered a variety of digital Stroop tasks (e.g., BrainBaseline, Encephalapp, M2C2, etc). We decided to use an eStroop that had been developed and piloted in our lab. Herein we report the comparability of this novel eStroop with a traditional paper version (pStroop).

**Method:**

Baseline neuropsychological assessments were conducted via videoconference on a study‐issued android tablet, with MCI patients and caregivers (*n* = 95; ave age = 72) before the start of the iPACES intervention. Executive function was assessed at baseline via videoconference, using a paper Stroop (pStroop, *n* = 40 stimuli: colored blocks, black text words, interference stimuli, timed by an administrator) and the self‐administered electronic Stroop (eStroop, *n* = 40 stimuli). In these analyses we examined the interference performances directly (e.g, Stroop C).

**Result:**

The pure interference trials of paper and digital measures of executive function: pStroop C and eStroop C, were found to correlate moderately (*r* = .59, *p* < .001).

**Conclusion:**

A novel digital biomarker, the interference portion of the electronic Stroop task (eStroop C), administered on a tablet to both MCI and caregivers, yielded comparable results to the interference portion of the traditional “paper” Stroop (pStroop C). Both tests were administered remotely, via videoconference as part of a baseline neuropsychological evaluation for an RCT initiated during the height of the COVID pandemic. Future clinical trials could consider adopting this self‐administered digital Stroop for a comparable measure of executive function, for ease of periodic administration.